# General Hospitalization and Intensive Care Unit-Related Factors of COVID-19 Patients in Northeastern Colombia: Baseline Characteristics of a Cohort Study

**DOI:** 10.7759/cureus.43888

**Published:** 2023-08-21

**Authors:** Catalina Cáceres Ramírez, Alvaro José Lora Mantilla, Laura Alejandra Parra Gómez, Valentina Ortegón Vargas, Mariam Posso Paz, Valeria Flórez Esparza, Edgar Gómez Lahitton, Silvia Juliana Villabona Flórez, Maria Catalina Rocha Lezama, Paul Anthony Camacho López

**Affiliations:** 1 Research, Development, and Technological Innovation Department, Fundación Oftalmológica de Santander, Floridablanca, COL

**Keywords:** colombia, signs and symptoms, cohort studies, covid-19, sars-cov-2

## Abstract

Objective

This study aims to describe demographic and clinical characteristics and the factors associated with the risk of COVID-19 general hospitalization and intensive care unit (ICU) care of patients who consulted in a third-level hospital in Santander, Colombia.

Methods

We used baseline data from an ambidirectional cohort study. We included all patients with positive real-time polymerase chain reaction (PCR) tests for COVID-19 who came to the emergency room (ER) for respiratory symptoms related to COVID-19. Information regarding patients' baseline characteristics and symptoms was collected through telephone interviews and review of medical records. Vital signs were extracted from medical records as well.

Results

We enrolled 3,030 patients, predominantly men, with a median age of 60 (interquartile range (IQR): 44-73). Symptoms of the acute phase varied between men and women. Men presented with more respiratory symptoms, and women had general symptoms. Hypertension, obesity, and diabetes were common risk factors for hospital admission. Antibiotic consumption may also play a role in hospital admission.

Conclusions

Male sex, older age, hypertension, obesity, prior thrombotic events, and self-medicated antibiotics were associated with general hospitalization. Hypertension, obesity, diabetes, and cancer were associated with ICU admission. The Charlson comorbidity index (CCI) is a powerful tool for evaluate the impact of pre-existing health conditions on COVID-19 hospital admission. We highlight the importance of these findings as possible predictors in our region.

## Introduction

On March 11, 2020, the World Health Organization (WHO) declared the COVID-19 pandemic with more than 761 million cases and 6.88 million deaths of patients due to COVID-19 confirmed globally [1]. Globally, upper-middle-income countries had the most cumulative confirmed cases, followed by high-income countries [2]. Argentina, Chile, Panama, Brazil, and Colombia are the nations in South America with the greatest overall cumulative rate of cases. However, Mexico, Ecuador, Paraguay, and Colombia are the ones with the greatest death rates [2]. This measure demonstrates the nation's capability to respond to the COVID-19 epidemic. The first case in Colombia was confirmed on March 6, 2020, and by January 2023, there have been more than 6.3 million confirmed cases and more than 142,000 deaths due to COVID-19 [3].

The Colombian healthcare system is a mix of public and private entities and is composed by two types of coverage: the contributory regimen is designed for the employed population that brings economic contributions to the system, and the subsidized regimen is intended for individuals who cannot afford healthcare, and its health coverage is assumed by the contributory regimen, reaching a universal insurance of 99.6% [4,5]. Despite this design, the healthcare system remains a challenge and faces several barriers to accessing care that includes economic, geographic, cultural, and administrative [6]. 

Several studies have been carried out to determine the epidemiological and clinical traits of COVID-19. Few Colombian cohorts have already been conducted both in the capital city and at a national level. These studies reported that the primary factors associated with intensive care unit (ICU) admission were severe pneumonia, an increase in the National Early Warning Score (NEWS) 2, ischemic heart disease, invasive mechanical ventilation, and chronic obstructive pulmonary disease (COPD). Moreover, factors associated with mortality included age >65 years, chronic kidney disease (CKD), ICU admission, and an increase in the Charlson comorbidity index (CCI) [7-9]. At the national level, the overall survival rates were 100%, 98%, 97%, and 95% for days 1, 10, 20, and 30, respectively [8]. Due to Colombia's epidemiologic transition process with the increase of re-emerging infectious respiratory diseases, further research is required to improve public health policies and decisions [10]. Our aim was to summarize the demographic, clinical, and risk factors connected to the likelihood that patients who visited a tertiary care hospital in Colombia would require ICU or general hospital care for COVID-19.

## Materials and methods

Study design, setting, and participants

We present baseline data from an ambidirectional cohort study conducted at the Fundación Oftalmológica de Santander (FOSCAL) located in Floridablanca, Colombia, in the metropolitan area of Bucaramanga. Bucaramanga is the capital of the northeast province of the department of Santander, surrounded by multiple rural towns and situated 3,146 feet (959 m) above sea level. The metropolitan area is made up of Floridablanca, Girón, Piedecuesta, and Bucaramanga, with a total population of 1,224,257 inhabitants. Between March 29, 2020 and September 27, 2021, all patients who reported to the emergency room (ER) with respiratory symptoms and had positive real-time polymerase chain reaction (PCR) tests for COVID-19 were included; this test was available in Colombia on March 20, 2020 [11]. We excluded a few patients: (1) those who arrived at the ER without vital signs or who underwent triage/pre-admission but died before being admitted and evaluated by a doctor; (2) those who were diagnosed post-mortem (patients did not have medical records and the Habeas Data Law does not allow to obtain information of the patient to contact their families); (3) patients with incomplete medical histories (incomplete medical history refers to those medical records in which the absence or presence of any data that was investigated in the survey could not be determined); (4) those who declined to participate; and (5) those who could not be reached.

The Research Ethics Committee of the FOSCAL Clinic approved this study (approval number: 02895/2020), and electronic informed consent was obtained from all study participants.

Procedures and outcome data

A retrospective telephone interview and an electronic medical record review were used to obtain clinical data from patients who were still alive as entirely as feasible. For patients with positive PCR and who had passed away from COVID-19 or other causes, only the electronic medical records were used to gather data. Data were then managed using LimeSurvey [12] to reduce missing entries and enable real-time data validation and quality control. Physicians with appropriate training conducted interviews with all living participants, requesting full completion of all questions covering demographic and clinical features. We collected the participants' demographic characteristics (age, sex, education level, occupation, and socioeconomic status), clinical factors (comorbidities, smoking, exposure to biomass combustion, pharmacological history of comorbidities, symptoms attributed to COVID-19 infection, and vital and physical signs in the ER), and the need for hospitalization due to COVID-19. Regarding smoking, two variables were defined: smoker (patient who has ever consumed tobacco in their life, either in the past or currently) and current smoker (patient who actually smoked tobacco at the time of the study inclusion). Cross-examination was performed with the patients to validate the data obtained in the medical records and to complete any missing data about demographic and clinical features. In addition, we inquired about any self-medicated alternative therapies used by patients for the prevention or symptomatic management of COVID-19, which were used 30 days prior to the index consultation to the hospital because of COVID-19.

COVID-19 signs and symptom data were questioned and reviewed in the medical records. Clinical records were used to gather information on vital and physical signs and biometric measurements, such as height, weight, and body mass index (BMI) in the ER. Intercostal or supraclavicular retractions evaluated by the emergency physicians were used to define clinical respiratory effort.

Charlson comorbidity index

The CCI categorizes comorbidities that may increase mortality risk over the next decade. CCI is a weighted index that utilizes the number and severity of various diseases, including cardiovascular diseases, myocardial infarction, congestive heart failure, peripheral vascular disease, cerebrovascular accident, dementia, hemiplegia, COPD, connective tissue disorders, peptic ulcer disease, liver disease, diabetes mellitus, CKD, cancer, and AIDS [13]. It has been previously validated in a Colombian population using medical records [14].

For each patient, a CCI score was calculated based on the total weights linked with the patient's comorbidities. We also incorporated the age category by adding one (1) point for each decade beyond age 50. Higher CCI scores indicate a higher mortality risk and more severe comorbid conditions. Peptic ulcer disease was not included because it was not found in the medical reports and could be confused with gastritis [15].

The CCI score is classified into three categories: 1-2, low mortality risk in 10 years; 3-4, moderate mortality risk in 10 years; and ≥5, high mortality risk in 10 years, with 90%, 53%, and 21% estimated 10-year survival, respectively. However, we classified patients into two groups: scores of <3 (0, 1, and 2) and scores ≥3; i.e., the first group has a lower risk of mortality in the preceding 10 years, and the second one has a high mortality risk in the next 10 years [16].

Statistical methods

We used Stata statistical software version 15.1 (StataCorp., 2017, College Station, Texas: StataCorp LLC) to perform our analysis. Descriptive data for continuous variables are presented as mean with standard deviation for variables with normal distribution and as median with interquartile range for those without normal distribution and absolute values and percentages for categorical variables. The participants were categorized by the treatment location into outpatient care, general hospitalization, and ICU care. A linear binomial regression model-type log was used to estimate the risk ratio (RR) and 95% confidence interval (95% CI) for demographic and clinical characteristics and overall risk of hospitalization and ICU care. We performed a crude analysis without knowing confounding factors (sex and age) and another one (our model 1) controlling for confounding factors. Finally, with those variables that showed significant association in the binomial analysis, we performed a multinomial logistic regression analysis to obtain a final model that could predict with RRs the association between these and obtaining general hospitalization and ICU care. The plot of the RR distribution and its 95% CI was made using the "Forestplot" package in R version 4.1.3 (R Foundation for Statistical Computing, Vienna, Austria).

## Results

**Figure 1:**
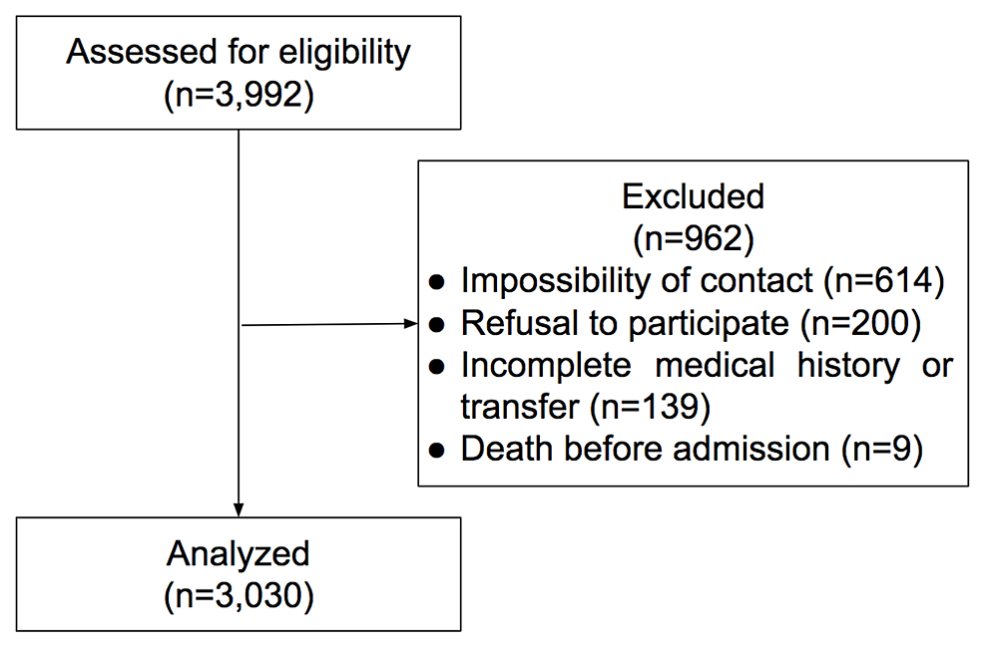
Consort diagram of the study

A total of 3,992 COVID-19 patients visited the ER. Of these, 962 patients were excluded: impossibility of contact (n= 614), refusal to participate (n= 200), incomplete medical history or transfer to another institution (n= 139), and death before admission (n= 9) (see Figure 1). Finally, a total of 3,030 patients were successfully enrolled.

The demographic and clinical characteristics of the participants at the entry of the study are shown in Table 1. The participants were predominantly males (1551 (51.2%) males vs. 1479 (48.8%) females), and the median age was 60 years (IQR: 44-73); men were older than women (median age of 62 vs. 58 years). Of the total, 996 (32.9%) patients were dead due to COVID-19 at the time of enrollment; men had higher mortality than women (39.7% vs. 25.6%). 

**Table 1:** Bivariate analysis of the influence of participants’ characteristics on the need for general hospitalization and ICU care of enrolled patients in the FOSCAL clinic between March 29, 2020, and September 27, 2021. RR: risk ratio, CKD: chronic kidney disease, COPD: chronic obstructive pulmonary disease, OSAHS: obstructive sleep apnea-hypopnea syndrome, MBP: median blood pressure

	RR general hospitalization (95% CI)	P	RR ICU care (95% CI)	P-value
Sex				
Female	Ref.		Ref.	
Male	1.31 (1.25-2.74)	<0.001	1.78 (1.58-2.00)	<0.001
Age, years (median (± IQR))				
18-30 years	Ref.		Ref.	
31-60 years	3.32 (2.73-4.05)	<0.001	4.84 (3.19-7.34)	<0.001
61-79 years	4.24 (3.49-5.16)	<0.001	7.21 (4.78-10.89)	<0.001
>80 years	4.17 (3.42-5.08)	<0.001	3.63 (2.31-5.70)	<0.001
Healthcare worker	0.09 (0.06-0.12)	<0.001	0.06 (0.03-0.13)	<0.001
Symptoms				
Cough	1.20 (1.14-1.27)	<0.001	1.40 (1.22-1.61)	<0.001
Dyspnea	1.97 (1.84-2.12)	<0.001	2.86 (2.42-3.38)	<0.001
Asthenia/adynamia	1.13 (1.08-1.18)	<0.001	1.17 (1.04-1.31)	0.011
Fever	1.28 (1.22-1.34)	<0.001	1.31 (1.17-1.48)	<0.001
Myalgia	0.97 (0.92-1.01)	0.170	0.93 (0.82-1.05)	0.240
Headache	0.77 (0.72-0.81)	<0.001	0.61 (0.52-0.70)	<0.001
Odynophagia	0.75 (0.70-0.80)	<0.001	0.81 (0.70-0.94)	0.004
Shivers	1.04 (0.98-1.09)	0.181	0.90 (0.78-1.04)	0.145
Anosmia	0.79 (0.74-0.84)	<0.001	0.64 (0.54-0.75)	<0.001
Diarrhea	1.11 (1.06-1.17)	<0.001	1.09 (0.95-1.25)	0.213
Ageusia	0.82 (0.76-0.88)	<0.001	0.61 (0.51-0.73)	<0.001
Arthralgia	0.94 (0.89-1.00)	0.063	0.91 (0.77-1.06)	0.215
Rhinorrhea	0.67 (0.62-0.73)	<0.001	0.66 (0.55-0.80)	<0.001
Chest pain	1.07 (1.01-1.14)	0.021	1.08 (0.91-1.27)	0.391
Emesis	1.03 (0.95-1.11)	0.495	0.86 (0.68-1.07)	0.170
Abdominal pain	0.94 (0.85-1.03)	0.163	0.77 (0.60-1.00)	0.046
Dizziness	1.00 (0.70-1.44)	0.995	0.31 (0.05-2.04)	0.225
Hemoptysis	1.30 (1.20-1.41)	<0.001	1.06 (0.65-1.74)	0.821
Epistaxis	1.11 (0.92-1.34)	0.277	0.93 (0.49-1.76)	0.828
Burning pain	0.56 (0.34-0.93)	0.024	0.31 (0.08-1.18)	0.085
Paresthesia	0.82 (0.58-1.18)	0.287	0.69 (0.29-1.66)	0.410
Seizures	1.07 (0.83-1.37)	0.599	0.96 (0.46-2.03)	0.918
Rash	0.87 (0.56-1.37)	0.554	0.63 (0.18-2.20)	0.469
Comorbidities				
Hypertension	1.41 (1.36-1.48)	<0.001	1.49 (1.34-1.67)	<0.001
Obesity	1.26 (1.21-1.31)	<0.001	1.59 (1.42-1.79)	<0.001
Diabetes	1.10 (1.09-1.12)	<0.001	1.16 (1.11-1.22)	<0.001
Hypothyroidism	1.18 (1.13-1.24)	<0.001	1.15 (1.00-1.32)	0.057
Dyslipidemia	1.25 (1.19-1.30)	<0.001	1.03 (0.88-1.19)	0.736
CKD	1.11 (1.09-1.15)	<0.001	1.09 (1.00-1.20)	0.060
Cancer	1.02 (0.98-1.06)	0.349	0.87 (0.76-0.99)	0.031
Ischemic heart disease	1.23 (1.17-1.30)	<0.001	1.38 (1.15-1.66)	0.001
COPD	1.23 (1.15-1.30)	<0.001	0.79 (0.60-1.05)	0.109
Heart failure	1.21 (1.14-1.30)	<0.001	0.83 (0.61-1.12)	0.220
Rhinitis	0.57 (0.47-0.69)	<0.001	0.26 (0.15-0.46)	< 0.001
Vasculopathy	1.19 (1.11-1.29)	<0.001	1.04 (0.78-1.39)	0.790
Asthma	0.82 (0.70-0.96)	0.016	0.72 (0.49-1.06)	0.095
Stroke	1.12 (1.02-1.23)	0.018	0.60 (0.39-0.92)	0.019
Rheumatic disease	1.10 (0.99-1.22)	0.075	1.11 (0.82-1.50)	0.500
Prior thrombotic event	1.22 (1.12-1.33)	<0.001	0.95 (0.65-1.40)	0.805
OSAHS	1.18 (1.02-1.36)	0.029	1.39 (0.93-2.09)	0.106
Charlson comorbidity index				
≥3 points	1.43 (1.37-1.49)	<0.001	1.37 (1.22-1.53)	<0.001
Pharmacological history				
Antihypertensive	1.37 (1.32-1.44)	<0.001	1.34 (1.20-1.50)	<0.001
Analgesic	0.93 (0.88-0.98)	0.013	0.86 (0.75-0.99)	0.005
Glucose-lowering	1.32 (1.27-1.37)	<0.001	1.39 (1.22-1.58)	<0.001
Lipid-lowering	1.27 (1.22-1.32)	<0.001	1.10 (0.96-1.26)	<0.001
Levothyroxine	1.19 (1.14-1.25)	<0.001	1.15 (0.99-1.33)	<0.001
Antibiotic	1.17 (1.12-1.23)	<0.001	0.99 (0.85-1.17)	<0.001
Proton pump inhibitor	1.25 (1.20-1.30)	<0.001	1.18 (1.02-1.37)	0.029
Corticoid	1.28 (1.23-1.34)	<0.001	1.34 (1.17-1.55)	<0.001
Antiplatelet	1.21 (1.16-1.27)	<0.001	1.17 (1.00-1.35)	0.043
Anticoagulant	1.29 (1.23-1.35)	<0.001	1.15 (0.96-1.37)	0.130
Ivermectin	0.62 (0.33-1.19)	0.153	0.94 (0.36-2.48)	0.909
Cigarette smoking				
Smoker	1.17 (1.12-1.23)	<0.001	0.99 (0.84-1.17)	0.898
Current smoker	0.67 (0.51-0.88)	0.004	0.90 (0.52-1.57)	0.710
Secondhand smoker	1.06 (0.97-1.16)	0.200	0.56 (0.39-0.80)	0.002
Smoking index (≥5)	1.24 (1.08-1.42)	0.002	1.22 (0.73-2.04)	0.459
Smoking index (≥10)	1.20 (1.06-1.36)	0.003	1.18 (0.78-1.80)	0.437
Exposure to biomass combustion				
	1.20 (1.13-1.27)	<0.001	0.64 (0.49-0.84)	0.001
Physical signs in the emergency room				
Capillary refill > 3 seconds	1.00 (0.96-1.05)	0.992	0.86 (0.76-0.96)	0.010
Respiratory effort	1.41 (1.37-1.45)	<0.001	2.21 (1.98-2.47)	<0.001
Rhinorrhea	0.54 (0.43-0.68)	<0.001	0.71 (0.49-1.03)	0.069
Vital signs in the emergency room				
MBP (≤60 mmHg)	1.20 (1.08-1.33)	0.001	0.69 (0.39-1.20)	0.184
Heart rate (≥100)	1.20 (1.15-1.25)	<0.001	1.21 (1.08-1.35)	0.001
Respiratory rate (≥20)	1.66 (1.58-1.79)	<0.001	2.18 (1.92-2.47)	<0.001
Oxygen saturation (≤90%)	1.87 (1.77-1.96)	<0.001	2.40 (2.06-2.79)	<0.001
Oxygen saturation (≤80%)	1.85 (1.76-1.95)	<0.001	4.17 (3.62-4.81)	<0.001
Body mass index				
<25 kg/m^2^	Ref.		Ref.	
25-29.9 kg/m^2^	1.33 (1.22-1.46)	<0.001	1.53 (1.16-2.01)	0.003
30-34.9 kg/m^2^	1.53 (1.39-1.66)	<0.001	2.51 (1.87-3.35)	<0.001
≥35 kg/m^2^	1.65 (1.49-1.84)	<0.001	3.10 (2.25-4.26)	<0.001

Data on the socioeconomic status were available for 2,212 patients; the majority belonged to the medium (1,310 (59.6%)) and low socioeconomic levels (750 (33.9%)). One-hundred thirty-one (4.3%) patients came from rural areas, and 25 (0.8%) were migrants. Likewise, the educational level of 2,090 patients was available, with COVID-19 being more frequent in patients with higher education (741 (35.4%) high school and 764 (36.5%) postgraduate). Data on the occupation were available for 2,835 patients; 345 (12.2%) were healthcare workers. Of these, 152 (48.1%) were nursing assistants, 73 (23.1%) were professional nurses, and 23 (7.2%) were general practitioners.

The most frequent symptom was cough, present in 2,124 patients (70.1%), followed by dyspnea (65.9%), asthenia/adynamia (62.2%), and fever (57.4%). Men and women experience the symptoms differently, with men more likely to experience dyspnea (71.9%), fever (63.1%), and hemoptysis (2%) than women. Females, on the other hand, were more likely to experience headaches (34.3%), myalgia (30.4%), odynophagia (25.7%), anosmia (22.8%), and ageusia (21%) (Table 1).

In the final study sample, the most frequent comorbidities were arterial hypertension in 1,237 patients (40.8%), obesity in 658 patients (21.7%), and diabetes in 619 patients (20.4%). Furthermore, the distribution of comorbidities was not equal between sexes; in men, hypertension (44.3%), obesity (22.4%), diabetes (23.3%), dyslipidemia (17.5%), CKD (10.3%), ischemic heart disease (8.9%), and heart failure (5.4%) prevailed. However, in females, hypothyroidism (21.8%), rhinitis (6.1%), asthma (4.7%), and rheumatic diseases (4%) were observed. Regarding CCI results, the majority of patients (56.5%) had a score of <3; furthermore, 80.3%, 47.7%, and 48.8% of the patients received ambulatory care, general hospitalization, and ICU admission, respectively. Finally, some patients were not under treatment for hypertension, diabetes, or dyslipidemia. A significant proportion of patients reported taking analgesics (22.5%), antibiotics (13.9%), and ivermectin (4.8%) for COVID-19 (Table 2).

**Table 2:** Characteristics of enrolled patients in the FOSCAL Clinic between March 29, 2020 and September 27, 2021. IQR: interquartile range, CKD: chronic kidney disease, COPD: chronic obstructive pulmonary disease, OSAHS: obstructive sleep apnea-hypopnea syndrome, SBP: systolic blood pressure, SD: standard deviation, DBP: diastolic blood pressure, MBP: median blood pressure

	Total (n=3,030)	Ambulatory care (n=824)	General hospitalization (n=1331)	ICU care (n=875)
Sex				
Female	1479 (48.8%)	551 (66.9%)	623 (46.8%)	305 (34.8%)
Male	1551 (51.2%)	273 (33.1%)	708 (53.2%)	570 (65.1%)
Age, years (median (±IQR))	60 (±29)	37 (±29)	65 (±24)	60 (±18)
18-30 years	386 (12.7%)	305 (37%)	59 (4.4%)	22 (2.5%)
31-60 years	1160 (38.2%)	351 (42.6%)	489 (36.7%)	320 (36.6%)
61-79 years	1107 (36.5%)	121 (14.7%)	531 (39.9%)	455 (52.0%)
>80 years	377 (12.4%)	47 (5.7%)	252 (18.9%)	78 (8.9%)
Death	996 (32.9%)	94 (11.4%)	326 (24.5%)	576 (65.8%)
Healthcare worker	345 (12.2%)	321 (39.7%)	18 (1.4%)	6 (0.8%)
Symptoms				
Cough	2124 (70.1%)	496 (60.2%)	957 (71.9%)	671 (76.7%)
Dyspnea	1997 (65.9%)	249 (30.2%)	1007 (75.6%)	741 (84.7%)
Asthenia/adynamia	1884 (62.2%)	450 (54.6%)	859 (64.5%)	575 (65.7%)
Fever	1740 (57.4%)	343 (41.6%)	838 (62.9%)	559 (63.9%)
Myalgia	895 (29.5%)	259 (31.4%)	391 (29.4%)	245 (28%)
Headache	879 (29.0%)	352 (42.7%)	353 (26.5%)	174 (19.9%)
Odynophagia	706 (23.3%)	296 (35.9%)	237 (17.8%)	173 (19.7%)
Shivers	655 (21.6%)	165 (20.0%)	316 (23.7%)	174 (19.9%)
Anosmia	606 (20.0%)	243 (29.5%)	243 (18.3%)	120 (13.7%)
Diarrhea	578 (19.1%)	120 (14.5%)	279 (20.9%)	179 (20.4%)
Ageusia	565 (18.6%)	217 (26.3%)	241 (18.1%)	107 (12.2%)
Arthralgia	508 (16.7%)	156 (18.9%)	217 (16.3%)	135 (15.4%)
Rhinorrhea	499 (16.5%)	241 (29.2%)	157 (11.8%)	101 (11.5%)
Chest pain	364 (12%)	82 (9.9%)	170 (12.7%)	112 (12.8%)
Emesis	248 (8.2%)	63 (7.6%)	123 (9.2%)	62 (7.1%)
Abdominal pain	212 (7.0%)	67 (8.1%)	97 (7.3%)	48 (5.5%)
Dizziness	189 (6.2%)	62 (7.5%)	87 (6.5%)	40 (4.6%)
Red eyes	54 (1.8%)	20 (4.3%)	28 (2.1%)	6 (0.7%)
Hemoptysis	36 (1.1%)	2 (0.2%)	23 (1.7%)	11 (1.2%)
Epistaxis	26 (0.9%)	5 (0.6%)	14 (1.0%)	7 (0.8%)
Burning pain	22 (0.7%)	13 (1.6%)	7 (0.5%)	2 (0.2%)
Paresthesia	20 (0.6%)	8 (1.0%)	8 (0.6%)	4 (0.4%)
Seizures	18 (0.6%)	4 (0.5%)	9 (0.7%)	5 (0.6%)
Rash	11 (0.4%)	4 (0.5%)	5 (0.4%)	2 (0.2%)
Comorbidities				
Hypertension	1237 (40.8%)	146 (17.7%)	647 (48.6%)	444 (50.7%)
Obesity	658 (21.7%)	87 (10.5%)	303 (22.7%)	268 (30.6%)
Diabetes	619 (20.4%)	55 (6.7%)	325 (24.4%)	239 (27.3%)
Hypothyroidism	501 (16.5%)	83 (10.1%)	256 (19.2%)	162 (18.5%)
Dyslipidemia	488 (16.1%)	62 (7.5%)	282 (21.2%)	144 (16.4%)
CKD	235 (7.8%)	25 (3.0%)	130 (9.8%)	80 (9.1%)
Cancer	224 (7.4%)	57 (6.9%)	117 (8.8%)	50 (5.7%)
Ischemic heart disease	198 (6.5%)	23 (2.8%)	98 (7.3%)	77 (8.8%)
COPD	168 (5.5%)	20 (2.4%)	109 (8.2%)	39 (4.4%)
Heart failure	137 (4.5%)	17 (2.0%)	87 (6.5%)	33 (3.8%)
Rhinitis	142 (4.7%)	82 (9.9%)	49 (3.7%)	11 (1.2%)
Vasculopathy	110 (3.6%)	15 (1.8%)	62 (4.6%)	33 (3.6%)
Asthma	100 (3.3%)	40 (4.8%)	39 (2.9%)	21 (2.4%)
Stroke	102 (3.4%)	19 (2.3%)	65 (4.9%)	18 (2.0%)
Rheumatic disease	94 (3.1%)	19 (2.3%)	45 (3.4%)	30 (3.4%)
Prior thrombotic event	69 (2.3%)	8 (1.0%)	42 (3.1%)	19 (2.2%)
OSAHS	33 (1.6%)	5 (0.9%)	14 (1.7%)	14 (2.3%)
Charlson comorbidity index				
<3 points	1715 (56.6%)	662 (80.3%)	1053 (47.7%)	427 (48.8%)
≥3 points	1315 (43.4%)	162 (19.7%)	1153 (52.3%)	448 (51.2%)
Pharmacological history				
Antihypertensive	1187 (39.2%)	149 (18.1%)	632 (47.5%)	406 (46.4%)
Analgesic	681 (22.5%)	212 (25.7%)	294 (22.1%)	175 (20.0%)
Glucose-lowering	547 (18.0%)	50 (6.1%)	292 (21.9%)	205 (23.4%)
Lipid-lowering	589 (19.4%)	71 (8.6%)	335 (25.2%)	183 (20.9%)
Levothyroxine	472 (15.6%)	74 (9.0%)	245 (18.4%)	152 (17.5%)
Antibiotic	421 (13.9%)	70 (8.5%)	230 (17.3%)	121 (13.8%)
Proton pump inhibitor	408 (13.5%)	49 (5.9%)	223 (16.7%)	136 (15.5%)
Corticoid	407 (13.4%)	41 (5.0%)	215 (16.1%)	151 (17.3%)
Antiplatelet	432 (14.3%)	62 (7.5%)	228 (17.1%)	142 (16.2%)
Anticoagulant	288 (9.5%)	25 (3.0%)	169 (12.7%)	94 (10.7%)
Ivermectin	146 (4.8%)	36 (4.4%)	81 (6.1%)	29 (3.3%)
Cigarette smoking				
Smoker (currently or past)	402 (13.3%)	66 (8%)	221 (16.6%)	115 (13.1%)
Current smoker	38 (9.4%)	16 (24.2%)	12 (5.4%)	10 (8.5%)
Secondhand smoker	156 (5.1%)	36 (4.4%)	94 (7.0%)	26 (3.0%)
Smoking index (median (±IQR))	6.45 (±18.5)	1.5 (±14.6)	7.5 (±19.6)	9.9 (±17.6)
Smoking index (≥5 to <10)	58 (18.0%)	6 (9.8%)	36 (20%)	16 (19.7%)
Smoking index (≥10)	123 (38.2%)	16 (26.2%)	74 (41.1%)	33 (40.7%)
Exposure to biomass combustion				
Exposure to biomass combustion	241 (7.9%)	34 (4.1%)	161 (12.1%)	46 (5.3%)
Physical signs in the emergency room				
Capillary refill > 3 seconds	1306 (44.4%)	349 (44.3%)	612 (46.8%)	345 (40.6%)
Respiratory effort	432 (14.7%)	11 (1.4%)	187 (14.3%)	234 (27.6%)
Rhinorrhea	111 (3.7%)	66 (8.4%)	22 (1.7%)	23 (2.7%)
Vital signs in the emergency room				
SBP, mmHg (mean (±SD))	131 (±20.4)	128.1 (±17.2)	130.6 (±21.2)	134.1 (±21.1)
DBP, mmHg (mean (±SD))	72 (±13.1)	73.1 (±11.2)	71 (±13.8)	71.1 (±13.4)
MBP, mmHg (mean (±SD))	91.6 (±13.9)	91.5 (±11.7)	90.9 (±14.7)	92.8 (±14.2)
MBP (≤60 mmHg)	50 (1.7%)	6 (0.8)	34 (2.6%)	10 (1.2%)
Heart rate (median (±IQR))	90 (±24)	85 (±21)	92 (±27)	18.8 (±24)
Heart rate (≥100)	1002 (34.3%)	173 (22.7%)	502 (38.5%)	327 (38.8%)
Respiratory rate (median (±IQR))	20 (±6)	18 (±3)	22 (±6)	24 (±6)
Respiratory rate (≥20)	1416 (48.8%)	103 (13.6%)	745 (57.2%)	568 (67.5%)
Oxygen saturation (median (±IQR))	91 (±11)	97 (±3)	89 (±9)	86 (±15)
Oxygen saturation (≤90% to ≥81%)	952 (32.7%)	33 (4.3%)	587 (45%)	332 (39.3%)
Oxygen saturation (≤80%)	494 (17.0%)	21 (2.7%)	173 (13.3%)	300 (35.5%)
Temperature (median (±IQR))	36.4 (±0.9)	36.2 (±0.7)	16.4 (±1)	36.4 (±1)
Body mass index				
<25 kg/m^2^	613 (31.5%)	301 (44.8%)	247 (26.5%)	65 (19%)
25-29.9 kg/m^2^	846 (43.5%)	273 (40.6%)	436 (46.7%)	137 (40.2%)
30-34.9 kg/m^2^	335 (17.2%)	74 (11%)	172 (18.4%)	89 (26.1%)
≥35 kg/m^2^	152 (7.8%)	24 (3.6%)	78 (8.4%)	50 (14.6%)

Outpatient care was more frequent in women and ICU care in men (F: 66.9% vs. M: 33.1% and F: 34.8% vs. M: 65.1%). Males had 1.31 (95% CI 1.25-1.37) more risk of needing available hospitalization care and 1.78 (95% CI 1.58-2.01) more ICU care needs than females, regardless of age. 

Based on COVID-19 symptoms, the most common symptom in outpatients was cough (60.2%), followed by asthenia/adynamia (54.6%) and headache (42.7%). However, in patients with general hospitalization and ICU care, the top three symptoms were dyspnea (75.6% / 84.7%), cough (71.9%/76.7%), and asthenia/adynamia (64.5%/65.7%). In outpatients, upper respiratory tract and general symptoms were more frequent; in patients with general hospitalization and ICU care, lower respiratory tract symptoms were the most frequent.

Dyspnea presented an RR of 1.59 (95% CI 1.50-1.70) for general hospitalization and 2.26 (95% CI 1.92-2.67) for ICU care. In the multivariate analysis, controlling confounding factors, such as age and sex, the most important predictive symptoms of general hospitalization were cough, dyspnea, fever, diarrhea, asthenia/adynamia, chest pain, and hemoptysis. However, for ICU care, the only associated symptoms were cough, dyspnea, fever, and chest pain. Moreover, some symptoms, such as odynophagia, rhinorrhea, burning pain, dizziness, ageusia, anosmia, and headache, were considered "protective" or at significantly lower risk for general hospitalization and ICU care (Table 3).

**Table 3:** Multivariate analysis of the enrolled patients in the FOSCAL Clinic between March 29, 2020 and September 27, 2021; controlling confounding factors (sex and age). RR: risk ratio, CKD: chronic kidney disease, COPD: chronic obstructive pulmonary disease, OSAHS: obstructive sleep apnea-hypopnea syndrome, MBP, median blood pressure

	RR general hospitalization (95% CI)	P	RR ICU care (95% CI)	P-value
Sex				
Female	Ref.		Ref.	
Male	1.31 (1.25-1.37)	<0.001	1.78 (1.58-2.01)	<0.001
Age, years (median (±IQR))				
18-30 years	Ref.		Ref.	
31-60 years	3.28 (2.69-3.99)	<0.001	4.41 (2.91-6.68)	<0.001
61-79 years	4.03 (3.32-4.89)	<0.001	6.37 (4.22-9.62)	<0.001
>80 years	4.03 (3.31-9.90)	<0.001	3.32 (2.12-5.21)	<0.001
Healthcare worker	0.13 (0.09-0.19)	<0.001	0.10 (0.05-0.23)	<0.001
Symptoms				
Cough	1.11 (1.07-1.16)	<0.001	1.30 (1.15-1.48)	<0.001
Dyspnea	1.59 (1.50-1.70)	<0.001	2.26 (1.92-2.67)	<0.001
Asthenia/adynamia	1.09 (1.05-1.13)	<0.001	1.10 (0.99-1.24)	0.082
Fever	1.13 (1.09-1.17)	<0.001	1.17 (1.05-1.31)	0.004
Myalgia	1.02 (0.98-1.05)	0.378	0.97 (0.86-1.09)	0.629
Headache	0.96 (0.92-1.01)	0.093	0.73 (0.63-0.84)	<0.001
Odynophagia	0.94 (0.90-0.99)	0.014	0.96 (0.84-1.10)	0.589
Shivers	1.04 (1.00-1.08)	0.031	0.92 (0.80-1.05)	0.217
Anosmia	0.96 (0.91-1.01)	0.120	0.75 (0.64-0.90)	0.001
Diarrhea	1.07 (1.03-1.10)	<0.001	1.08 (0.95-1.23)	0.266
Ageusia	0.98 (0.93-1.03)	0.486	0.70 (0.59-0.84)	<0.001
Arthralgia	0.96 (0.74-1.24)	0.763	0.91 (0.68-1.21)	0.541
Rhinorrhea	0.89 (0.84-0.95)	<0.001	0.84 (0.71-0.99)	0.050
Chest pain	1.54 (1.13-2.12)	0.006	1.58 (1.12-2.23)	0.009
Emesis	1.00 (0.93-1.06)	0.904	0.94 (0.76-1.16)	0.554
Abdominal pain	0.94 (0.88-1.02)	0.152	0.80 (0.62-1.02)	0.074
Dizziness	1.00 (0.71-1.40)	0.994	0.62 (0.39-0.98)	0.042
Hemoptysis	1.15 (1.05– 1.26)	0.003	0.96 (0.60-1.53)	0.879
Epistaxis	1.35 (0.44-4.15)	0.592	0.96 (0.27-3.40)	0.957
Burning pain	0.59 (0.037– 0.95)	0.028	0.34 (0.09-1.24)	0.103
Paresthesia	0.75 (0.52-1.08)	0.126	0.43 (0.10-1.76)	0.242
Seizures	1.08 (0.87-1.34)	0.569	0.93 (0.21-3.94)	0.922
Rash	0.94 (0.67-1.34)	0.747	0.50 (0.07-3.24)	0.474
Comorbidities				
Hypertension	1.07 (1.03-1.11)	<0.001	1.19 (1.06-1.34)	0.003
Obesity	2.87 (2.16-3.80)	<0.001	1.46 (1.31-1.63)	<0.001
Diabetes	1.02 (1.00-1.03)	0.003	2.59 (1.86-3.61)	<0.001
Hypothyroidism	1.01 (0.97– 1.05)	0.448	1.06 (1.01-1.11)	0.018
Dyslipidemia	1.02 (0.98-1.06)	0.379	0.84 (0.72-0.97)	0.021
CKD	1.00 (0.97-1.02)	0.806	1.00 (0.92-1.10)	0.935
Cancer	0.94 (0.92-0.98)	0.004	0.80 (0.70-0.90)	<0.001
Ischemic heart disease	0.99 (0.95-1.05)	0.964	1.13 (0.95-1.35)	0.173
COPD	1.02 (0.97-1.08)	0.397	0.76 (0.58-1.01)	0.062
Heart failure	0.99 (0.93-1.05)	0.833	0.75 (0.56-1.00)	0.051
Rhinitis	0.81 (0.68-0.95)	0.011	0.41 (0.23-0.71)	0.001
Vasculopathy	1.00 (0.93-1.07)	0.994	0.92 (0.69-1.21)	0.539
Asthma	0.97 (0.86-1.11)	0.720	0.89 (0.61-1.29)	0.531
Stroke	0.94 (0.86-1.03)	0.197	0.38 (0.19-0.76)	0.006
Rheumatic disease	0.98 (0.55-1.74)	0.965	1.07 (0.57-1.98)	0.830
Prior thrombotic event	1.16 (1.09-1.25)	<0.001	0.87 (0.60-1.26)	0.460
OSAHS	1.00 (0.88-1.14)	0.957	1.21 (0.483– 1.73)	0.309
Charlson comorbidity index				
≥3 points	1.34 (1.29-1.40)	<0.001	1.29 (1.16-1.45)	<0.001
Pharmacological history				
Antihypertensive	1.04 (1.00-1.08)	0.015	1.06 (0.95-1.19)	0.296
Analgesic	0.98 (0.94-1.02)	0.480	0.93 (0.82-1.06)	0.295
Glucose-lowering	1.06 (1.03-1.09)	<0.001	1.08 (0.95-1.22)	0.242
Lipid-lowering	1.02 (0.99-1.06)	0.113	0.90 (0.78-1.02)	0.104
Levothyroxine	1.02 (0.98-1.06)	0.195	1.16 (1.01-1.34)	0.033
Antibiotic	1.06 (1.03-1.10)	0.001	0.91 (0.77-1.06)	0.214
Proton pump inhibitor	1.06 (1.02-1.09)	0.001	1.04 (0.90-1.20)	0.574
Corticoid	1.08 (1.05-1.11)	<0.001	1.16 (1.01-1.33)	0.026
Antiplatelet	1.00 (0.97-1.04)	0.670	0.99 (0.86-1.15)	0.963
Anticoagulant	1.06 (1.02-1.10)	<0.001	1.02 (0.86-1.21)	0.815
Ivermectin	0.57 (0.31-1.07)	0.081	0.87 (0.35-2.12)	0.765
Cigarette smoking				
Smoker (currently or past)	1.02 (0.98-1.06)	0.395	0.61 (0.43-0.86)	0.006
Current smoker	0.82 (0.67-1.03)	0.091	0.43 (0.15-1.15)	0.095
Secondhand smoker	1.06 (0.98– 1.15)	0.148	0.68 (0.38-1.21)	0.197
Smoking index (5-9)	1.23 (1.09-1.38)	0.001	1.09 (0.65-1.83)	0.726
Smoking index (≥10)	1.16 (1.03-1.30)	0.016	1.11 (0.73-1.69)	0.620
Exposure to biomass combustion				
Exposure to biomass combustion	1.04 (0.99-1.08)	0.092	0.61 (0.48-0.80)	<0.001
Physical signs in the emergency room				
Capillary refill > 3 seconds	0.98 (0.95-1.01)	0.203	0.85 (0.76-0.95)	0.007
Respiratory effort	1.28 (1.23-1.32)	<0.001	2.00 (1.79-2.24)	<0.001
Rhinorrhea	0.78 (0.66-0.93)	0.007	1.04 (0.74-1.45)	0.825
Vital signs in the emergency room				
MBP (≤60 mmHg)	1,17 (1.09-1.25)	<0.001	0.67 (0.39-1.15)	0.145
Heart rate (≥100)	1.08 (1.06-1.12)	<0.001	1.15 (1.03-1.28)	0.015
Respiratory rate (≥20)	1.36 (1.30-1.42)	<0.001	1.77 (1.56– 2.00)	<0.001
Oxygen saturation (90%-81%)	1.54 (1.47-1.61)	<0.001	1.96 (1.69-2.28)	<0.001
Oxygen saturation (≤80%)	1.53 (1.45-1.60)	<0.001	3.22 (2.79-3.72)	<0.001
Body mass index				
<25 kg/m^2^	Ref.		Ref.	
25-29.9 kg/m^2^	1.26 (1.16-1.38)	<0.001	1.34 (1.02-1.75)	0.035
30-34.9 kg/m^2^	1.43 (1.30-1.57)	<0.001	2.15 (1.62-2.85	<0.001
≥35 kg/m^2^	1.47 (1.33-1.63)	<0.001	2.64 (1.95-3.60)	<0.001

Patient's comorbidities and pharmacological and toxicological history were crucial for the COVID-19 disease and its evolution. Hypertension, obesity, and prior thrombotic events were related to general hospitalization for COVID-19. However, receiving ICU care was associated with hypertension, obesity, diabetes, and cancer. Regarding CCI, a score ≥3 points was associated with general hospitalization (RR = 1.34, 95% CI 1.29-1.4) and ICU care (RR = 1.29, 95% CI 1.16-1.45). Some pharmacological histories were related to a higher risk of being hospitalized, such as glucose-lowering, antibiotic, proton pump inhibitor, corticoids, and anticoagulants. In toxicological history, focused on pulmonary disease risk factors, we found that in our patients, being a smoker or second-hand smoker was not related to getting hospital admission.

Moreover, a critical part of the physician's decision-making is the patient's state at the time of the consultation in the ER; we found a significant relationship between having respiratory effort at the consultation time and getting hospitalized (RR = 1.28; 95% CI 1.23-1.32) and ever higher with getting ICU care (RR = 2.00; 95% CI 1.79-2.24). Moreover, vital signs are critical in the first patient evaluation in the emergency room; having a heart rate upper than 100 beats per minute, respiratory rate upper than 20 breaths per minute, and lower oxygen saturation (≤90% and 80%) were related with general hospitalization and ICU care need. In addition, BMI greater than 25 kg/m^2^ showed a significant relationship between with getting hospitalized (RR = 1.26; 95% CI 1.16-1.38) and getting ICU care (RR = 1.34; 95% CI 1.02-1.75).

In Appendix A, the final model with the significant variables of the multivariate analysis of participant characteristics with the need for general hospitalization and ICU care is presented. It is observed that being male, age, hypertension, tachycardia, and oxygen saturation below 90% are associated with these two outcomes. In addition, diabetes, prior use of antibiotics, corticosteroids, and anticoagulants, as well as a BMI above 25, are associated with general hospitalization. Obesity and respiratory distress are associated with admission to the ICU.

**Figure 2:**
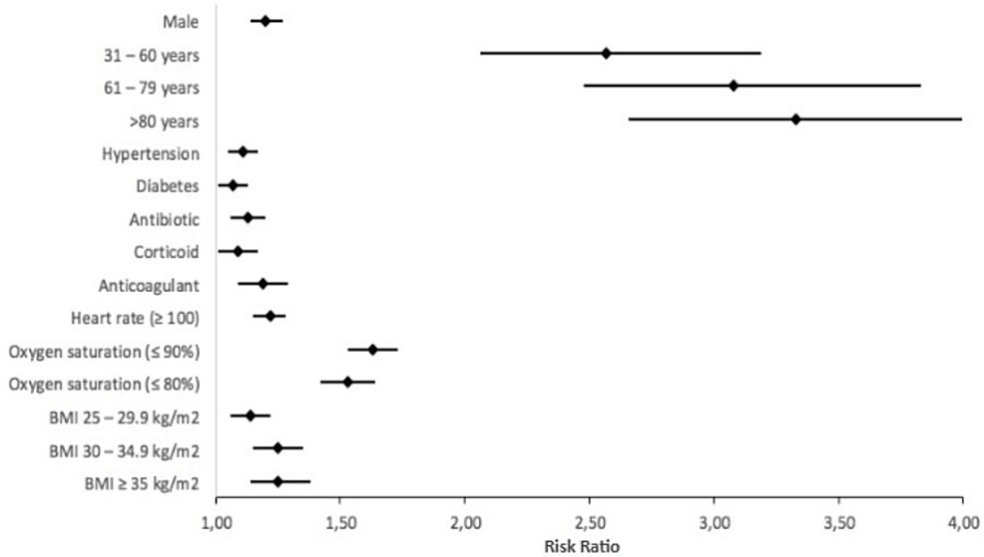
Forest plot of the general hospitalization risk ratio.

Finally, we evaluated a prediction model for getting hospitalized or getting ICU care; we included variables that were significant in the multivariate analysis by controlling the confounding factors of sex and age, and in the final model, we had just the variables that were still significant at the inclusion of the model. The variables associated with hospitalization (general or ICU care) were being male, age ≥31 years old, hypertension, obesity, respiratory effort, heart rate ≥100 beats per minute, and oxygen saturation ≤ 90% (Figure 2, Table 4).

**Table 4:** Multivariate analysis of the influence of the participants’ characteristics on the need for general hospitalization and ICU care of enrolled patients in the FOSCAL Clinic between March 29, 2020 and September 27, 2021. Final model with the significant variables. RR: risk ratio

	RR General hospitalization (CI 95%)	P	RR ICU care (CI 95%)	P-value
Sex				
Female	Ref.		Ref.	
Male	1.20 (1.14-1.27)	<0.001	1.41 (1.26-1.58)	<0.001
Age, years				
18-30 years	Ref.		Ref.	
31-60 years	2.57 (2.06-3.19)	<0.001	2.66 (1.78-3.96)	<0.001
61-79 years	3.08 (2.48-3.83)	<0.001	3.27 (2.18-4.91)	<0.001
>80 years	3.33 (2.66-4.18)	<0.001	1.79 (1.14-2.81)	0.011
Comorbidities				
Hypertension	1.11 (1.05-1.17)	<0.001	1.18 (1.05-1.32)	0.004
Obesity			1.32 (1.17-1.47)	<0.001
Diabetes	1.07 (1.01-1.13)	0.015		
Pharmacological history				
Antibiotic	1.13 (1.06-1.20)	<0.001		
Corticoid	1.09 (1.01-1.17)	0.025		
Anticoagulant	1.19 (1.09-1.29)	<0.001		
Physical signs in the emergency room				
Respiratory effort			1.22 (1.08-1.37)	0.002
Vital signs in the emergency room				
Heart rate (≥100)	1.22 (1.15-1.28)	<0.001	1.29 (1.14-1.48)	<0.001
Oxygen saturation (≤90%)	1.63 (1.53-1.73)	<0.001	1.73 (1.47-2.04)	<0.001
Oxygen saturation (≤80%)	1.53 (1.42-1.64)	<0.001	2.65 (2.24-3.13)	<0.001
			Ref.	
			1.41 (1.26-1.58)	
			Ref.	
			2.66 (1.78-3.96)	
Body mass index				
<25 kg/m^2^	Ref.			
25-29.9 kg/m^2^	1.14 (1.06-1.22)	<0.001		
30-34.9 kg/m^2^	1.25 (1.15-1.35)	<0.001		
≥35 kg/m^2^	1.25 (1.14-1.38)	<0.001		

## Discussion

We summarize the demographic, clinical, and hospitalization-related parameters of 3,030 COVID-19 patients treated in a third-level hospital in Colombia. We specifically examined the factors associated with the risk of COVID-19 general hospitalization and ICU care. 

In our baseline, men were more likely to be admitted to general hospitalization and ICU than women. Other studies also converge with this finding, identifying that COVID-19 affects predominantly males and that mortality rate is higher in this population [17]. This finding was also reported in other Colombian cohorts in which men had higher risk of mortality, ICU admission, and mechanical ventilation [8,9]. Both behavioral and biological factors related to the immune response and the anti-inflamatorry and antithrombotic effects of estrogen may be responsibles for these events [18]. Estrogen is known to promote innate and adaptive immune responses, which leads to faster removal of the virus and fewer symptoms in the acute stage of the illness. In addition, it has the potential to decrease the expression of ACE2 receptors on type 2 alveolar epithelial cells, reduce the attachment between SARS-CoV-2 and ACE2 receptors, and inhibit the entry of SARS-CoV-2 into host cells [18]. Considering this, estrogens and ACE inhibitors could be a potential target for the tratment of other viral infections with a similar pathophysiology of COVID-19 [19]. Considering that smoking is more prevalent in men, active smoking correlates with a higher expression of ACE2 genes [20]. 

The mortality rates of different cohorts should be considered with caution. The healthcare setting where the patients were recruited implies an underlying illness severity that affects directly on this indicator. Our cohort was done in the emergency department and found a death rate of 32.8%, a figure lower than the reported in two big cohorts from Brazil and Peru that found a mortality of 50% and 46%, respectively [21,22], but they included patients hospitalized, representing higher severity of illness and augmented mortality rates. By contrast, an observational study conducted in Bogotá, Colombia, from March 2020 to April 2021, including patients of all healthcare settings, i.e., outpatient clinic, ER, hospitalization, and ICU care, found an older patient death rate of 25% [9]; however, the patients included in this cohort have a decreased prevalence of comorbidities that could influence a lower mortality. Other factors that could influence the mortality rates were the introduction of vaccination programs, the COVID-19 waves in different times between countries and cities, the ER availability of beds, and the socioeconomic status [23,24].

Hypertension was the most common comorbidity, followed by obesity and diabetes. These findings are supported by other studies, such as those in Argentine and New York cohorts, where the most common comorbidities were arterial hypertension and obesity [25,26]. We identified a greater prevalence of hypertension compared to a cohort of 5,161 adult COVID-19 patients from Bogotá, Colombia, which was 23% [9]. However, a significant study carried out by Camacho et al. reports a hypertension rate of 37% across Colombia's rural and urban populations, which is more similar to our data [27]. 

Hypertension leads to some changes that can affect the severity of COVID-19, such as dysregulation of the renin-angiotensin-aldosterone system, gastrointestinal dysfunction, and imbalance in inflammation and immune response [28]. Moreover, other authors found that hypertension is an independent factor on the severity and mortality of COVID-19, with an RR for death, disease severity, and the possibility of ICU admission of 1.79 (1.68-1.89 with 95% CI), 1.74 (1.66-1.83 with 95% CI), and 1.91 (1.48-2.34 with 95% CI), respectively [29]. 

Although few data are available on the relationship between patients’ BMI and COVID-19 infection, several studies have shown that obesity may be an important factor for the risk and outcomes of patients infected with SARS-COV-2, including Colombian studies [30]. Whether obesity is an independent risk factor for infection in our population requires further research, but it is clear that excess adipose tissue acts as a pro-inflammatory element, causing metabolic dysfunction that can lead to dyslipidemia, insulin resistance, type 2 diabetes mellitus, hypertension, and cardiovascular disease, which are major risk factors for disease severity and mortality associated with COVID-19 [31]. 

Peng et al. performed a retrospective analysis of 112 patients with COVID‐19 infection and observed that the BMI of the critical patient group was significantly higher than that of the general group (P = 0.003), and among the non‐survivor patients, 88.2% of them had a BMI > 25 kg/m^2^, which is significantly higher (P < 0.001) than in the survivors (18.9%) [32]. These findings were similar to those in a UK report of hospitalized COVID-19 patients in which obesity was associated with a higher mortality, despite finding a considerably low prevalence (11%) [33].

Although the CCI was first developed to predict mortality, other studies have also proven to be reliable to predict different outcomes in other populations [34]. That is why most studies linking COVID-19 to the CCI focus on predicting the risk of death, and their results are quite promising [35]. We identified a favorable correlation between a CCI score of ≥3 and the chance of general hospitalization and ICU treatment. According to a recent systematic study, a CCI score more than 0 is related to increased mortality in COVID-19 patients, with a 16% increase in mortality risk per point rise in the CCI score. This study also discovered a correlation between the CCI score and poor outcomes and illness severity, but not mechanical ventilation [36]. Another study found that the age-adjusted CCI in COVID-19 patients is an independent predictor of the requirement for mechanical ventilation and in-hospital mortality, using a cutoff of ≥4. However, it was not a reliable indication of hospital length of stay [37].

With clinical and demographic variables, and adjusting for confounding factors, we created a final predictive model for general hospitalization or ICU care in COVID-19 patients. This model includes male sex, age (≥31 years old), comorbidities (hypertension and obesity), and clinical factors (respiratory effort, heart rate ≥100 beats per minute, and oxygen saturation ≤90%). Our predictive model corresponds with the published by Ioannou et al. [38], which found male sex, age ≥50, and hypertension as risk factors for hospitalization for COVID-19. By contrast, in this cohort race (Black and Asian), urban population, underweight, diabetes, CKD, cirrhosis, alcohol dependence, and CCI ≥1 are also defined as risk factors for hospitalization. Clinical factors were not evaluated. Heo et al. developed a scoring system to predict patients requiring ICU care for COVID-19. In accord with our findings, age ≥31 and male sex are related to ICU admission. In addition, they found that body temperature at admission ≥37 °C, hemoptysis, dyspnea, CKD, and functional dependence are also related [39]. Using machine learning, Islam et al. [40] developed a prognostic model for ICU admission in patients with COVID-19, and they found older age and male sex as risk factors too, with the rest of variables associated with ICU care found to be correlational with radiological and paraclinical variables. In Brazil, Soares Rde et al. [41] published a cohort study in 2020 where age ≥60, male gender, cardiovascular disease, and obesity were found as risk factors for hospitalization, such as our cohort; in addition, they report race, diabetes, CKD, pulmonary disease, smoking, and clinical symptoms (fever and dyspnea) as other risk factors.

There was evidence of frequent use of self-medicated analgesics, antibiotics, and antiparasitic, specifically ivermectin, in our population for symptomatic relief. This phenomenon resulted from the numerous unfounded recommendations spread across the nation at the start of the pandemic as a preventative measure to slow the spread of the coronavirus [41,42]. Nevertheless, in other nations, the use of antibiotics was expected, and many doctors would prescribe them even in cases where there was no bacterial infection. Our research showed that the use of antibiotics was associated with hospital admission (RR = 1.06, CI 95% 1.03-2.10) but not ICU admission (RR = 0.91, CI 95% 0.77-1.06). A meta-analysis of 154 studies revealed that the estimated prevalence of bacterial coinfection was lower than the antibiotic prescription rate (antibiotic prescription 74.6%, 95% CI 68.3-80.0% vs. bacterial infection 8.6%, 95% CI 4.7-15.2%) [43]. A multicenter research in Colombia reported a prescription rate for antibiotics of 36.6% and a coinfection rate of 4.3% [44]. 

The use of biomass fuels (principally wood) is widely spread in developing countries for cooking and space heating, and its deleterious effect on climate, air quality, and human health is well known [45]. Several studies have discovered that biomass smoke exposure is linked to the development of COPD, particularly in rural women in developing nations [46,47]. In our study, we found an association of biomass exposure with general hospitalization (RR = 2.01, CI 95% 1.32-3.05). It has been shown that smokers and COPD patients have greater levels of ACE-2 expression in their lungs [48]. The association between smoking and worse outcomes related to COVID-19 infection has been documented in multiple studies [41,46]. However, in our multivariate analysis, we did not find an association between smoking (current/past) and the risk of hospitalization or ICU admission, despite having higher scores on the smoking index. Only the bivariate analysis showed a significant association between a smoking index ≥5 and overall hospitalization. This may be explained by the fact that 66% of the patients admitted to the ICU died, and data collection for them was only based on medical records, potentially leading to underreporting of this variable.

Limitations 

Our study may have a selection bias as we excluded patients with incomplete data or individuals who did not sign the informed consent. The information was collected through phone calls; thus, an information bias could be presented. We relied on physician criteria and skills to write the medical history. Moreover, we had a high rate of unresponsive patients. Although our team used to call several times, sometimes patients did not want to answer unknown numbers because they thought it could be a scam. 

In Colombia, the vaccination program was done in a five-stage process: starting with a rapid vaccination of health workers and elderly adults (≥60 years old) and finalizing with the general population being a lower a delayed process [49]. Approximately 49% of the patients of the cohort were included after the completion of the first people vaccinated scheme (healthcare workers and ≥60 years old), but only 52 patients (1.7%) were included after the completion of the general population stage of vaccination. The effect of the vaccines in patients under 60 years old is not significant in this analysis because during the data collection period, vaccine coverage in this population was very low. Therefore, there could be a non-differential information bias that leads to an underestimation of the potential risk of the variables under study.

Finally, we used a sample of patients that consulted the ER for COVID-19 symptoms. Thus, the clinical characteristics of patients who attended the ER could be more severe than those who did not. Nevertheless, the main strength of our study is the important number of patients we interviewed and obtained data from.

## Conclusions

This study examined COVID-19 patient data from a tertiary care hospital in Colombia, focusing on demographic, clinical, and risk factors influencing the need for ICU or general hospital care. Gender disparities are evident, with higher general and ICU hospitalization rates for men. Comorbidities, such as hypertension, obesity, and prior thrombotic events, contribute significantly to hospitalization risk as self-medicated antibiotics. Meanwhile, hypertension, obesity, diabetes, and cancer were associated with ICU admission. The inclusion of the CCI in this study carries crucial significance as it serves as a powerful tool for comprehensively evaluating the impact of pre-existing health conditions on COVID-19 outcomes.

Predictive models incorporating age, sex, and clinical indicators offer valuable tools for assessing patient needs. Despite potential biases and limitations, this study with an extensive sample provides substantial insights, guiding policy decisions and emphasizing the relevance of identified risk factors in shaping effective healthcare strategies for COVID-19 and other acute respiratory infections (ARI) in Colombia.

## Appendices

Appendix A 

**Table 5:** Multivariate analysis of the influence of the participants’ characteristics on the need for general hospitalization and ICU care of enrolled patients in the FOSCAL Clinic between March 29, 2020 and September 27, 2021. Final model with the significant variables. RR: risk ratio

	RR general hospitalization (CI 95%)	P	RR ICU care (CI 95%)		P-value
Sex					
Female	Ref.		Ref.		
Male	1.20 (1.14-1.27)	<0.001	1.41 (1.26-1.58)		<0.001
Age, years					
18-30 years	Ref.		Ref.		
31-60 years	2.57 (2.06-3.19)	<0.001	2.66 (1.78-3.96)		<0.001
61-79 years	3.08 (2.48-3.83)	<0.001	3.27 (2.18-4.91)		<0.001
>80 years	3.33 (2.66-4.18)	<0.001	1.79 (1.14-2.81)		0.011
Comorbidities					
Hypertension	1.11 (1.05-1.17)	<0.001	1.18 (1.05-1.32)		0.004
Obesity			1.32 (1.17-1.47)		<0.001
Diabetes	1.07 (1.01-1.13)	0.015			
Pharmacological history					
Antibiotic	1.13 (1.06-1.20)	<0.001			
Corticoid	1.09 (1.01-1.17)	0.025			
Anticoagulant	1.19 (1.09-1.29)	<0.001			
Physical signs in the emergency room					
Respiratory effort			1.22 (1.08-1.37)		0.002
Vital signs in the emergency room					
Heart rate (≥100)	1.22 (1.15-1.28)	<0.001	1.29 (1.14-1.48)		<0.001
Oxygen saturation (≤90%)	1.63 (1.53-1.73)	<0.001	1.73 (1.47-2.04)		<0.001
Oxygen saturation (≤80%)	1.53 (1.42-1.64)	<0.001	2.65 (2.24-3.13)		<0.001
Body mass index					
<25 kg/m^2^	Ref.				
25-29.9 kg/m^2^	1.14 (1.06-1.22)	<0.001			
30-34.9 kg/m^2^	1.25 (1.15-1.35)	<0.001			
≥35 kg/m^2^	1.25 (1.14-1.38)	<0.001			
